# Cortico-subthalamic Coherence in a Patient With Dystonia Induced by Chorea-Acanthocytosis: A Case Report

**DOI:** 10.3389/fnhum.2019.00163

**Published:** 2019-05-28

**Authors:** Chunyan Cao, Peng Huang, Tao Wang, Shikun Zhan, Wei Liu, Yixin Pan, Yiwen Wu, Hongxia Li, Bomin Sun, Dianyou Li, Vladimir Litvak

**Affiliations:** ^1^Department of Functional Neurosurgery, Affiliated Ruijin Hospital, School of Medicine, Shanghai JiaoTong University, Shanghai, China; ^2^Department of Neurology, Affiliated Ruijin Hospital, School of Medicine, Shanghai JiaoTong University, Shanghai, China; ^3^Wellcome Centre for Human Neuroimaging, UCL Queen Square Institute of Neurology, London, United Kingdom

**Keywords:** DBS, magnetoencephalography (MEG), human, movement disorder, oscillations

## Abstract

The subthalamic nucleus (STN) is a common target for deep brain stimulation (DBS) treatment in Parkinson’s disease (PD) but much less frequently targeted for other disorders. Here we report the results of simultaneous local field potential (LFP) recordings and magnetoencephalography (MEG) in a single patient who was implanted bilaterally in the STN for the treatment of dystonia induced by chorea-acanthocytosis. Consistent with the previous results in PD, the dystonia patient showed significant subthalamo-cortical coherence in the high beta band (28–35 Hz) on both sides localized to the mesial sensorimotor areas. In addition, on the right side, significant coherence was found in the theta-alpha band (4–12 Hz) that localized to the medial prefrontal cortex with the peak in the anterior cingulate gyrus. Comparison of STN power spectra with a previously reported PD cohort showed increased power in the theta and alpha bands and decreased power in the low beta band in dystonia which is consistent with most of the previous studies. The present report extends the range of disorders for which cortico-subthalamic oscillatory connectivity has been characterized. Our results strengthen the evidence that at least some of the subthalamo-cortical oscillatory coherent networks are a feature of the healthy brain, although we do not rule out that coherence magnitude could be affected by disease.

## Introduction

Synchronized oscillations are a prevalent phenomenon in neural systems and are hypothesized to play an important role in communication between different neuronal populations (Friston et al., 2015). Cortical oscillatory connectivity in the human brain can be studied non-invasively with electroencephalography (EEG) and magnetoencephalography (MEG; Gross, 2014). However, activity from subcortical nuclei can only be recorded invasively. Deep brain stimulation (DBS) surgery provides a unique opportunity to study the subcortical activities by recording local field potentials (LFPs) from macroelectrodes which are stereotactically targeted with high precision to specific anatomical structures. DBS is a powerful treatment for Parkinson’s disease (PD; Limousin et al., 1995), dystonia (Cao et al., 2013) and also for severe obsessive compulsive disorder (OCD; Chabardès et al., 2013; Mulders et al., 2016). The subthalamic nucleus (STN) is the most common DBS target, primarily used for PD. The internal Globus Pallidus (GPi) is the primary target for dystonia but it is also used in PD. Thus, while electrophysiological markers of PD have been well characterized in both STN and GPi, those of dystonia are mostly known from GPi recordings. A view consolidated in recent years sees both PD and dystonia as oscillopathies (Nimmrich et al., 2015), disorders closely linked to abnormal oscillatory activity in the cortico-basal ganglia circuits. For dystonia the abnormal activity is in theta-alpha (4–12 Hz) range (Silberstein et al., 2003); whereas, for PD the abnormality is in the beta (13–30 Hz) range (Brown et al., 2001). Several previous studies directly comparing LFP power spectra between the two disorders reproduced the same pattern with increased alpha-theta power in dystonia relative to PD and increased beta power in PD (particularly after withdrawal of dopaminergic medication) relative to dystonia. This pattern was observed in both GPi (Silberstein et al., 2003; Wang et al., 2018; Piña-Fuentes et al., 2019) and STN (Neumann et al., 2012; Geng et al., 2017). One study (Wang et al., 2016) did not find clear differences in STN LFP spectra between the dystonia and PD groups. In the motor cortex, the differences between dystonia and PD are less pronounced with one study showing an increase of the peak frequency of alpha and beta oscillations in PD relative to dystonia (Crowell et al., 2012) and another study finding increased broadband high gamma activity in PD but no differences for alpha and beta (Miocinovic et al., 2015). For both dystonia and PD, the study of abnormal oscillations can have clinical implications for improvement of DBS targeting (Yoshida et al., 2010; Horn et al., 2017; Neumann et al., 2017) and development of brain activity driven closed-loop DBS methods (Little et al., 2013; Barow et al., 2014; Meidahl et al., 2017; Piña-Fuentes et al., 2019).

The mechanisms that generate pathological rhythms are not well understood. The modeling work to date has focused primarily on the pathological beta in PD where several competing models have been put forward (see Pavlides et al., 2015 for review). The main unresolved question in modeling work, which is equally likely to apply to theta-alpha in dystonia, is whether the pathological synchronization is generated locally in the basal ganglia or involves abnormal amplification of cortical inputs. Simultaneous recording of MEG and LFP could potentially make it possible to distinguish between these theories as it facilitates the characterization of the interaction between subcortical activity seen in the LFP and cortical areas whose activity can be recorded by MEG (Harmsen et al., 2018).

The two published studies of simultaneous MEG and STN LFP recordings in PD patients at rest (Hirschmann et al., 2011; Litvak et al., 2011a), both show that the STN is coherent with the mesial motor areas [likely, the supplementary motor area (SMA)] in the beta range and with the temporo-parietal areas and the brainstem in the alpha-theta range. A study applying similar methods to dystonia patients with GPi electrodes (Neumann et al., 2015) found beta coherence with the sensorimotor cortex, theta coherence with the inferior temporal cortex and alpha coherence with the cerebellum. The latter was correlated with the degree of clinical impairment.

To determine the relation between these oscillatory coherent networks and pathophysiology of disease it would be helpful to compare recordings from the same DBS target for different neurological disorders. To this end, here we report here an analysis of STN-cortical coherence in a patient with dystonia. This combination of methods, target and disease has not been reported before.

## Materials and Methods

### Patient Details

The study was approved by the local ethics committee of Ruijin hospital, Shanghai JiaoTong University School of Medicine. The patient was informed about the aim and the scope of the study and gave written informed consent. The patient also consented to the publication of the present case report. A 34 years old male developed involuntary movements 4 years prior to the surgery and was diagnosed with chorea-acanthocytosis. Neuroacanthocytosis due to mutations in the VPS13A gene encoding chorein is an autosomal-recessive neurodegenerative disorder which is characterized by chorea and dystonia (Rampoldi et al., 2001). The patient presented with impairment of fine movements of the upper limbs and bradykinesia, mainly involving the right limb. In addition, the patient opened and closed his mouth involuntarily, snorted intermittently and gradually developed lower limb weakness. Two years before the surgery, the symptoms of the left limb gradually became more obvious and difficulty in swallowing and coughing from drinking water appeared. Blood tests showed red blood cell count of 5.25 × 10^12^/L. Some red blood cells varied in size and had a spiked cell membrane consistent with the appearance of acanthocytes. Genetic testing revealed a mutation in the gene VPS13A. The structural magnetic resonance imaging (MRI) showed lateral ventricle enlargement and putamen and caudate nucleus atrophy. The patient was not pharmacologically treated prior to surgery.

### Deep Brain Stimulation Operation and MEG-LFP Recording

Implantation of the quadripolar DBS electrodes (model 3387; Medtronic, Minneapolis, MN, USA) was performed under general anesthesia bilaterally using a MRI-guided targeting (3.0 T, General Electric), which was co-registered with a CT image (General Electric) with the Leksell stereotactic frame (Zhan et al., 2018). The electrode leads were externalized for a week and temporary externally applied stimulation during this period could partially control the patient’s symptoms particularly the involuntary movement of the mouth, pronunciation, and hand movement impairment. We cannot report the results of long-term follow-up at this stage since the patient was operated less than a year ago. We also used the externalization period to record bilateral STN-LFP and whole head MEG simultaneously while the patient was awake. The MEG was recorded with a 306-channel MEG scanner (Elekta Oy, Helsinki, Finland) in a magnetically shielded chamber (Euroshield, Eura, Finland). The EEG system which is integrated with the MEG device was used for the LFP recording. The patient was instructed to rest with eyes open, and the absence of voluntary movement was confirmed by continuous visual inspection. The raw data were band pass filtered in 0.03–330 Hz range and digitized at 1,000 Hz. The DBS electrode had four platinum-iridium cylindrical surfaces of diameter 1.27 mm, length 1.5 mm and center-to-center separation 1.5 mm. The contacts were numbered 0–3 from the ventral to dorsal.

### Reconstruction of Electrode Locations in the STN

We used the Lead-DBS toolbox^1^ (Horn and Kühn, 2015) to reconstruct the contact locations. Post-operative CT was co-registered to pre-operative T1 scan using a two-stage linear registration (rigid followed by affine) as implemented in Advanced Normalisation Tools (ANT^2^; Avants et al., 2008). The pre-operative T2 scan was linearly co-registered to pre-operative T1 using SPM12 (Friston et al., 2007). Pre- (and post-) operative acquisitions were spatially normalized into MNI_ICBM_2009b_NLIN_ASYM space (Fonov et al., 2011) using the SyN registration approach in ANT. DBS-Electrodes were automatically pre-localized in native and template space using the PaCER algorithm^3^ (Husch et al., 2018) and then manually localized based on post-operative acquisitions using a tool specifically designed for this task (as implemented in Lead-DBS software, Horn and Kühn, 2015).

### Analysis of LFP and MEG

**Figure 1:**
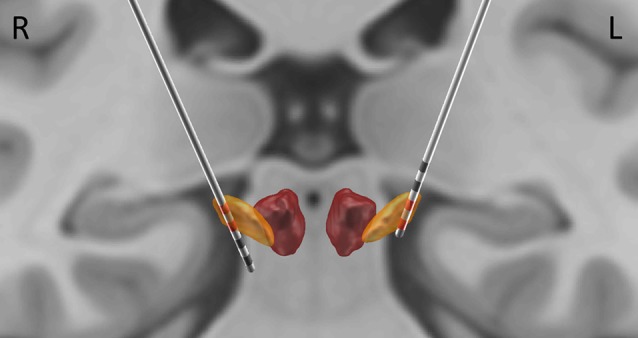
Localization of electrode contacts. The localization was performed based on post-operative CT, coregistered to pre-operative magnetic resonance imaging (MRI). Subthalamic nucleus (STN) and the Red Nucleus are visualized based on DISTAL atlas (Ewert et al., 2018). The contacts used for bipolar derivations reported here (2, 3 on the right; 0, 1 on the left) are highlighted in red.

MEG data were pre-processed with the temporal extension of Signal Space Separation method (Taulu and Simola, 2006) implemented in the MaxFilter software (Elekta Oy, Helsinki, Finland). The subsequent analyses were done in SPM12^4^ (Litvak et al., 2011b) following the procedures described by Litvak et al. (2010, 2011a). LFP data from contacts located in the STN (see Figure 1) were converted to bipolar montage which gave one LFP channel per side. The data were high-pass filtered above 1 Hz and 50 Hz line noise and its harmonics were removed with notch filters (Butterworth 5th order, zero phase filters). The data were epoched into 3 s segments. Epochs containing deflections exceeding 20 μV in the LFP channels were removed from analysis which left 136 epochs of clean LFP data which was visually verified.

LFP spectra were computed using multi-taper spectral estimation method (Thomson, 1982). The power was averaged across trials, subjected to log-transform and normalized by subtracting a linear fit to the 55–95 Hz range from the whole spectrum. The aim of these steps was to be able to compare the spectra to our previous recordings done on different system (see below).

Significant cortico-STN coherence was identified by statistical comparison of scalp-frequency images of coherence in the 1–45 Hz range to surrogate images generated by shuffling the reference channel across trials. This procedure was described in detail by Litvak et al. (2011a). The identified significant coherence patterns were source localized for the corresponding frequency band and reference channel using Dynamic Imaging of Coherent Sources (DICS) beamforming (Gross et al., 2001) implemented in the Data Analysis in Source Space (DAiSS) toolbox for SPM^5^. This used a single shell forward model (Nolte, 2003) generated based on the patient’s pre-operative T1 using standard SPM procedures (Mattout et al., 2007).

### Comparison to PD Cohort

In order to examine the similarities and differences in STN spectra between the patient reported here and previously studied PD patients, we compared the results to those from a cohort previously reported by Litvak et al. (2011a). All the patients reported in the article were included except for patient 3 who was not responsive to dopaminergic medication.

## Results

**Figure 2:**
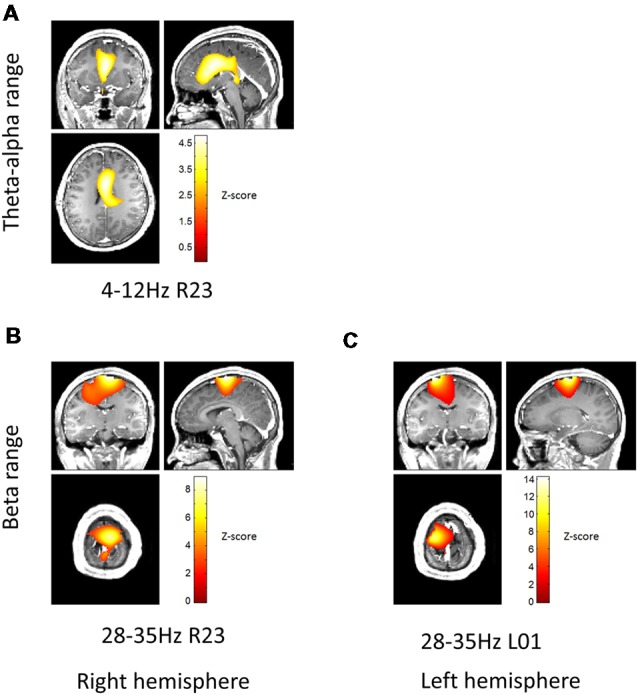
Beamformer localization of significant coherent clusters. The frequency bands for each local field potentials (LFPs) channel were determined based on sensor-level test (see “Materials and Methods” section). Dynamic Imaging of Coherent Sources (DICS) coherence beamformer (Gross et al., 2001) was used for source analysis. For visualization purposes, the beamformer images were converted to *Z*-score across voxels, thresholded above 2.5 and overlaid on the patient’s pre-operative T1 structural. Panel **(A)** shows localization of theta-alpha (4–12 Hz) coherence with the right STN. Panels **(B,C)** show localization of high beta (28–35 Hz) coherence with the right and left STN, respectively.

The top two contacts (2 and 3) of the right electrode were localized inside the right STN; the bottom two contacts (0 and 1) of the left electrode were localized inside the left STN (Figure 1). The sensor level analysis revealed three significant clusters of coherence. We only report the frequency bands obtained from the sensor-level analysis here and Figure 2 shows the corresponding source localizations. On the right side, two clusters were identified. The first cluster was in the theta-alpha band (4–12 Hz, Figure 2A) and localized to the deep frontal medial areas with the peak in the anterior cingulate cortex. The second cluster was in the beta band (28–35 Hz, Figure 2B) and localized to the ipsilateral pre-motor and motor cortices. A similar single beta cluster was found for the left STN channel with the same significant frequency range (Figure 2C) and localization to the corresponding areas on the left side.

**Figure 3:**
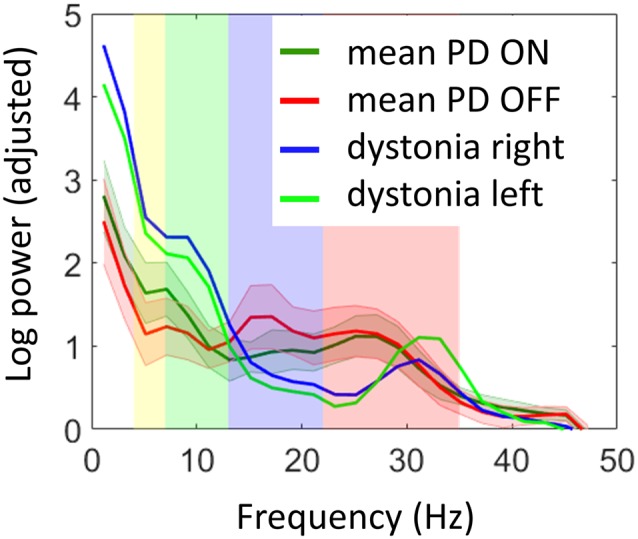
Comparison of STN power spectra of the dystonia patient with the previously reported Parkinson’s disease (PD) cohort (Litvak et al., 2011a). Log power was normalized by subtracting a linear fit to the spectrum in the 55–95 Hz range. The shaded error bars denote 95% confidence intervals for the mean of PD spectra. The background color shows the standard electrophysiological frequency bands: theta (yellow, 4–7 Hz), alpha (green, 7–13 Hz), low beta (purple, 13–22 Hz) and high beta (pink, 22–35 Hz). The dystonia patient showed increased power in theta and alpha bands and reduced power in low beta (see “Results” section for details).

Comparison of LFP power spectra to the previous PD cohort showed higher alpha-theta power for the dystonia patient and much lower power in the low beta range when compared to both ON and OFF dopamine states in PD (Figure 3). There was a distinct peak in power in the high beta band corresponding to the range of significant coherence between STN and the cortex which exceeded the mean values in this range for PD. In order to determine whether there were any significant differences between the dystonia patient and the PD cohort, we performed two sample *t*-tests between the two hemispheres of the dystonia patient and 22 hemispheres of the PD patients OFF drug assuming equal variance for power spectra averaged in pre-defined bands (shown in color in Figure 3). For theta (4–7 Hz) and alpha (7–13 Hz) the results were close to significance (M_PD_ = 1.15, M_dyst_ = 2.45, SD = 0.88, *t* = −2.01, *p* = 0.057 and M_PD_ = 1.12, M_dyst_ = 2.07, SD = 0.64, *t* = −2.01, *p* = 0.058 respectively) but without correction for multiple testing across bands. For low beta (13–22 Hz) and high beta (22–35 Hz) the differences were not significant (M_PD_ = 1.2, M_dyst_ = 0.68, SD = 0.66, *t* = 1.07, *p* = 0.29 and M_PD_ = 0.96, M_dyst_ = 0.66, SD = 0.6, *t* = 0.68, *p* = 0.5, respectively).

## Discussion

The present case report reproduces some of the previous findings regarding abnormal low-frequency oscillatory activity in dystonia and adds to the range of conditions for which cortico-subthalamic interactions have been studied with concurrent LFP-MEG recordings. It is now possible to discuss the commonalities and differences between PD (Hirschmann et al., 2011; Litvak et al., 2011a), OCD (Wojtecki et al., 2017) and dystonia. This gives a larger degree of confidence when speculating about the expected patterns of connectivity in the healthy state which cannot be assessed with invasive recording techniques.

### LFP Power Spectra

The features of LFP power spectra we found are consistent with the majority of previous studies. Geng et al. (2017) reported a direct group comparison of STN LFP in dystonia and PD and their findings were very similar to ours: increased low-frequency power and reduced beta power in dystonia compared to PD. Neumann et al. (2012) report similar results for a single case. However, Wang et al. (2016), did not find significant differences between the two patient populations. Low-frequency activity has been shown to be a hallmark of dystonia in several studies of DBS patients with recordings in the GPi (Barow et al., 2014; Neumann et al., 2017). Furthermore, low-frequency power increase in the STN LFP was associated with induction of dyskinesia by dopaminergic drugs in PD (Alonso-Frech et al., 2006). Thus, it could be the case that this feature is associated with the symptom of hyperkinetic movements rather than a particular disease. Silberstein et al. (2003) compared pallidal LFP recorded intraoperatively between PD and dystonia and found exactly the same pattern as reported here: the 4–10 Hz power is highest in dystonia, intermediate in PD on medication and lowest in PD off medication; 11–30 Hz power is highest in PD off medication, intermediate in PD on medication and lowest in dystonia. Low beta power has been demonstrated as a robust biomarker of clinical impairment induced by PD, particularly bradykinesia and rigidity. The symptoms of the patient reported here included right limb bradykinesia. However, the low beta power in the contralateral (left) STN was below that of the ipsilateral side. We, therefore, suggest that bradykinesia in dystonia might not necessarily be associated with increased low beta power in the STN. The left STN did show increased high beta power compared to the right STN but that was largely above the frequency range where beta power is increased in PD patients. High-beta power increase in the GPi was recently linked to hyperkinetic symptoms in Huntington’s disease (Zhu et al., 2018) so beta activity could also be related to the patient’s choreatic mouth movements. In order to determine whether and how high beta power increase is related to disease symptoms, observations in more patients are necessary.

### Coherence in Dystonia, OCD and PD

Coherence in the high beta band (22–35 Hz) is common to all the studies of cortico-subthalamic interactions published to date (Lalo et al., 2008; Hirschmann et al., 2011; Litvak et al., 2011a; Oswal et al., 2016; Wojtecki et al., 2017; Belardinelli et al., 2019). In all the MEG studies where source localization was possible the cortical coherent sources localized to the ipsilateral mesial sensorimotor areas. These findings are also in line with simultaneous intraoperative cortical surface and STN LFP recordings (Whitmer et al., 2012). The cortical areas consistently shown as being coherent with the STN LFP in this band correspond well to those where the hyperdirect cortico-STN pathway is known to originate in humans (Lambert et al., 2012). The fact that high beta coherence is present in all the three clinical conditions: PD, dystonia and OCD suggests that it is likely to be present also in the healthy state. Whether and how the magnitude of high beta coherence is affected by disease is still an open question.

Our finding of theta-alpha coherence between the STN and anterior cingulate is very similar to the finding in OCD reported by Wojtecki et al. (2017). The frequency range of this coherence overlaps with that of theta-alpha power increase compared to the PD cohort. The two phenomena could, therefore, be related. Low frequency coherence between the STN and medial prefrontal cortex has been the subject of a number of recent studies where it has been shown to be involved in adjustment of decision thresholds for high conflict trials during decision-making tasks (Zavala et al., 2014, 2016, 2018; Herz et al., 2017; Hell et al., 2018; Kelley et al., 2018). Interestingly, despite the fact that all these studies were done on PD patients with STN-DBS, low-frequency coherence between the STN and medial prefrontal cortex was not observed at rest in PD (Hirschmann et al., 2011; Litvak et al., 2011a) and instead both studies reported coherence with temporo-parietal areas in the same band with Litvak et al. also reporting additional peak in the brainstem. There could be several possible explanations for these discrepancies. It could be the case that they reflect the differences between the three disorders. In that case, it would be likely that STN-prefrontal resting coherence is suppressed in PD because it is present in both OCD and dystonia. Alternatively, the differences could be due to different placement of the electrodes in the STN. This nucleus is commonly divided into motor, associative and limbic part which have different connectivity with the cortex (Lambert et al., 2012).

### Limitations of the Study

To put the results of the single case recording reported here in context, we compare them to those from the previously reported PD patient cohort. However, it should be noted that those recordings were done using a different LFP amplifier. Although the spectra were normalized to minimize any contribution of the hardware differences and the results are in line with what could be expected, a comparison of LFPs recorded with the same hardware would, of course, be preferable. It would also be interesting to compare the coherence magnitude of the dystonia patient with the PD cohort but comparing the typical coherence values for patients from our cohort recorded on a CTF MEG system with those reported in the PD literature for the Neuromag system (Hirschmann et al., 2011, 2013) raised a suspicion that there is a systematic difference with Neuromag yielding lower values. We, therefore, opted to not include such a comparison in the present report as it could be misleading. Unfortunately, PD patients with STN electrodes are not available at our site for clinical reasons.

## Conclusion

The present report extends the range of disorders for which cortico-subthalamic oscillatory connectivity has been studied. Our results support the suggestion that oscillatory coherent networks are not solely features of disease. However, whether these networks are affected by the disease and whether their modulation is causally related to disease pathophysiology remains an open question.

## Ethics Statement

The study was approved by the local ethics committee of Ruijin hospital, Shanghai JiaoTong University, School of Medicine. The patient was informed about the aim and the scope of the study and gave written informed consent. The patient also consented to the publication of the present case report.

## Author Contributions

PH and TW did the MEG recording and collected clinical data. YW and HL recorded performed clinical video recordings. BS, SZ, WL, DL and YP performed the DBS surgery. CC and VL analyzed the data and wrote the article.

## Conflict of Interest Statement

The authors declare that the research was conducted in the absence of any commercial or financial relationships that could be construed as a potential conflict of interest.

The reviewer AM declared a shared affiliation, though no other collaboration, with one of the authors VL to the handling Editor.

## Footnotes

^1^http://www.lead-dbs.org/

^2^http://stnava.github.io/ANTs/

^3^http://adhusch.github.io/PaCER/

^4^https://www.fil.ion.ucl.ac.uk/spm/software/

^5^https://github.com/spm/daiss
